# Early Triassic ichthyopterygian fossils from the Russian Far East

**DOI:** 10.1038/s41598-022-09481-6

**Published:** 2022-04-01

**Authors:** Yasuhisa Nakajima, Yasunari Shigeta, Alexandra Houssaye, Yuri D. Zakharov, Alexander M. Popov, P. Martin Sander

**Affiliations:** 1 Department of Natural Sciences, Faculty of Science and Engineering, Tokyo City University, 1-28-1 Tamazutsumi, Setagaya-ku, Tokyo, 158-8557 Japan; 2 Department of Geology and Paleontology, National Museum of Nature and Science, 4-1-1 Amakubo, Tsukuba, Ibaraki 305-0005 Japan; 3 Département Adaptations du Vivant, UMR 7179 CNRS/Muséum National d’Histoire Naturelle, 57 rue Cuvier CP-55, 75005 Paris, France; 4 Far Eastern Geological Institute, Russian Academy of Sciences, Far Eastern Branch, Stoletiya Prospect 159, Vladivostok, Russia 690022; 5 Section Paleontology, Institute of Geosciences, University of Bonn, 53115 Bonn, Germany

**Keywords:** Palaeontology, Stratigraphy

## Abstract

Ichthyopterygia is a major clade of reptiles that colonized the ocean after the end-Permian mass extinction, with the oldest fossil records found in early Spathian substage (late Olenekian, late Early Triassic) strata in the western USA. Here, we describe reptilian remains found in situ in the early Spathian *Neocolumbites insignis* ammonoid zone of South Primorye in the Russian Far East. Specimen NSM PV 23854 comprises fragmentary axial elements exhibiting a combination of morphological characteristics typical of Ichthyopterygia. The cylindrical centra suggest that the specimen represents a basal ichthyopterygian, and its size is comparable to that of *Utatsusaurus*. Specimen NSM PV 24995 is represented by a single limb bone, which is tentatively identified as an ichthyopterygian humerus. With a body length of approximately 5 m estimated from the humeral length, NSM PV 24995 represents one of the largest specimens of early Spathian marine reptiles known to date. Such size variation among the earliest ichthyopterygians might suggest an explosive diversification in size immediately after the end-Permian mass extinction. Both vertebrae and humerus specimens exhibit an extremely cancellous inner structure, suggesting a high degree of aquatic adaptation in ichthyopterygians, despite their short history of evolution in the ocean.

## Introduction

**Figure 1:**
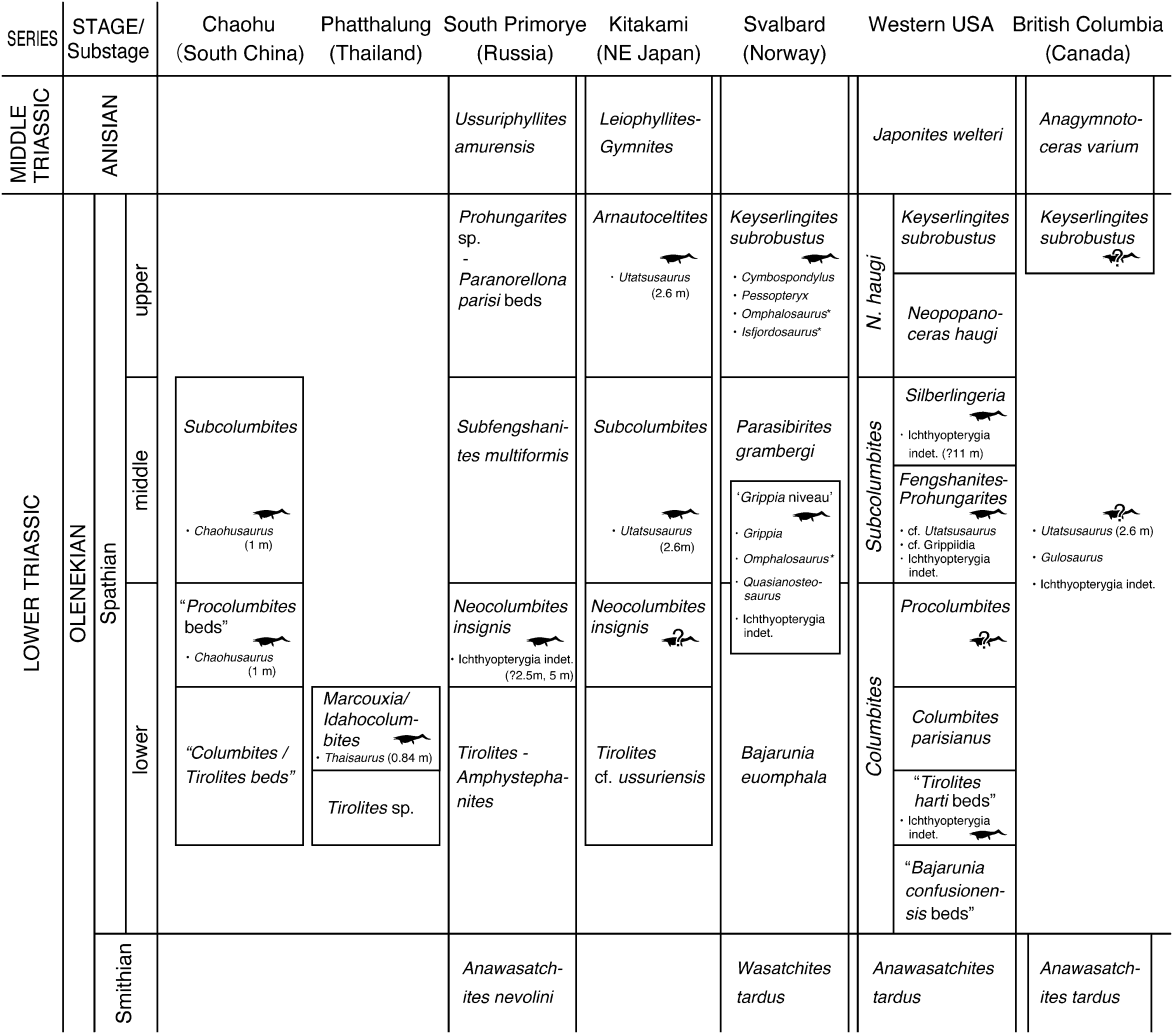
Ammonoid biostratigraphy of Spathian (Olenekian, Lower Triassic) ichthyopterygian and possible ichthyopterygian fossil localities. Ammonoid biostratigraphic data were compiled after Ji et al.^7^ for Chaohu (South China); Tongtherm et al.^5^ for Phatthalung (Thailand); Zakharov et al.^20,22^ and Shigeta and Kumagae^73^ for South Primorye (Russia); Bando and Shimoyama^59^, Ehiro et al.^61^, Shigeta and Nakajima^29^, and Shigeta^60^ for Kitakami (Northeast Japan); Hurum et al.^11^ and Hansen et al*.*^12^ for Svalbard (Norway); Guex et al.^56^ and Brayard et al.^55^ for the Western United States; Tozer^57^ for British Columbia (Canada). Ichthyopterygian horizons are also compiled after Ji et al.^7^ for Chaohu; Tongtherm et al.^4^ for Phatthalung; Shikama et al*.*^32^ for Kitakami; Maxwell and Kear^10^, Hurum et al*.*^11^, Hansen et al*.*^17^, Engelschiøn et al.^18^, and Ekeheien et al.^16^ for Svalbard; Massare and Callaway^14^, Scheyer et al*.*^2^, Kelley et al.^15^, Smith et al*.*^3^ for Western USA; Brinkman et al.^38^ and Cuthbertson et al.^12,39^ for British Columbia. Silhouettes indicate the occurrence of ichthyopterygian fossils. Possible source of materials without reliable ammonoid biostratigraphic information is indicated with a question mark. Taxonomic names are given in generic or higher rank with approximate body length when available. Asterisks indicate disputed taxa. The *Grippia* Niveau is likely from either *Bajarunia euomphala* or *Parasibirites gramberg*i zone.

Multiple clades of Reptilia entered the ocean following the end-Permian mass extinction to become new members of Mesozoic marine ecosystems. Ichthyopterygia represent a clade of marine reptiles that are highly adapted to an aquatic lifestyle and include the fish-shaped reptile Ichthyosauria^1^. Ichthyopterygia first appear in the fossil record in the Spathian substage (late Olenekian, late Early Triassic), within 5 million years of the Permian–Triassic (P–T) mass extinction event^2^. The oldest known ichthyopterygian material is a vertebral centrum from *Tirolites* ammonoid biozone (lower lower Spathian, ~ 3 myr after the P–T boundary) in NoName locality of northeastern Nevada^3^. *Thaisaurus chonglakmanii*, a small ichthyopterygian from the Phukhaothong Dolomite Member of the Chaiburi Formation in the Phatthalung area, southern Thailand^4^, is one of the oldest named taxon of Mesozoic marine reptiles; the *Thaisaurus* horizon belongs to the middle lower Spathian^5^ (Fig. 1), which is slightly younger than NoName locality centrum. *Chaohusaurus* spp. are small (approximately 1 m long) ichthyopterygians found in the *Procolumbites* and *Subcolumbites* ammonoid zones (upper lower–middle Spathian) of the Majiashan locality near Chaohu Lake, China^6,7^. Recent research has revealed that close relatives of Ichthyopterygia, that is, basal members of the more inclusive clade Ichthyosauromorpha, lived alongside *Chaohusaurus*^1,7^. Later, Ichthyopterygia became cosmopolitan marine predators in the Early Triassic^8^. Before the end of the Early Triassic, small to midium ichthyopterygians (body length < 3.0 m) such as *Chaohusaurus*, *Grippia*, *Gulosaurus*, and *Utatsusaurus* appeared in many localities around the Northern Hemisphere, including British Columbia of Canada, southern China, Svalbard of Norway, the western United States, and northeastern Japan^2,7,9–12^ (Fig. 1).

As an exceptional case, a 28-cm-long “ichthyosaur humerus” (NMMNH P-65886) was discovered in the *Silberlingeria* subzone of the upper part of the *Subcolumbites* zone of the Thaynes Formation in Bear Lake County, Idaho, Western USA (Fig. 1)^2^. The estimated total body length of this animal would have been approximately 11 m^2^, although Jiang et al.^13^ questioned these taxonomical and anatomical identifications and suggested that this body size estimate should be revised downward^13^. Even if NMMNH P-65886 is not an ichthyopterygian humerus but a coracoid of an aquatic reptile, as Jiang et al.^13^ suggested, this animal still be huge for an Early Triassic marine vertebrate. Fragments of ichthyopterygians, which are possibly even older, were found in the same country as surface floats from the Thaynes Formation, with the most probable source rock being the lower black shale member (Smithian to lower Spathian^14^). One of the Massare and Callaway^14^ specimens (IMNH 38918), representing the distal portion of the left humerus, is similar in shape to that of the Middle Triassic ichthyosaur *Cymbospondylus petrinus* (*C. piscosus*) but approximately 3/4 of the size^14^, and might represent a 7-m-class ichthyosaur. However, the age estimate of these fragments is less reliable than that of NMMNH P-65886. Small to medium-sized ichthyopterygian jaw fragments including cf. *Utatsusaurus* and cf. Grippiida have been reported from *Prohungarites* subzone of Lower Member, Prida Formation in Fossil Hill, Nevada^15^.

The Lower Triassic lower Vendomdalen Member of the Vikinghøgda Formation in central Spitzbergen, Norway, includes the horizon referred to as ‘*Grippia* niveau.’ This bonebed yields ichthyopterygian fossils, most of which are assigned to small ichthyopterygians including *Grippia* and larger *Quasianosteosaurus*, plus a disputed ichthyopterygian *Omphalosaurus*^10,16^. *Grippia* niveau is dated as the lower Spathian (*Bajarunia euomphala* zone) or middle Spathian (*Parasibirites grambergi* zone)^17^ (Fig. 1). From Lower Saurian niveau (upper Spathian, *Keiserlingites subrobustus* zone) of the same member in the Marmierfjellet of Spitzbergen, many other fragmentary reptilian fossils including large ichthyopterygians (*Pessopteryx*, *Cymbospondylus* and disputed *Isfjordosaurus*) and *Omphalosaurus*^10,18^ are found.

As mentioned above, recent studies have gradually elucidated the diversity of early Mesozoic marine reptiles. However, the paleobiogeographic information of marine reptiles through the Early Triassic is still scant. Moreover, the early stage of evolution toward the pelagic lifestyle in Ichthyopterygia is poorly understood. Here, we report the occurrence of two ichthyopterygian specimens: NSM (National Museum of Nature and Science, Tokyo) PV 23854 and NSM PV 24995, from the Lower Triassic strata of Cape Zhitkov, Russky Island, in the Russian Far East. With detailed biostratigraphic data, these specimens were collected in situ from the lower Spathian *Neocolumbites insignis* zone, which is regarded as the same age as the *Procolumbites* beds in southern China, from which the oldest known specimen of *Chaohusaurus* was reported^6,7,19^. The large size and three-dimensional preservation of the new skeletal elements would help to reveal the process of gigantism and aquatic adaptation in Ichthyopterygia based on functional histology.

## Materials

### Institutional abbreviations

NSM, National Museum of Nature and Science, Tokyo, Japan; LACM, Natural History Museum of Los Angeles County, Los Angeles, California, USA, NMMNH, New Mexico Museum of Natural History and Science, Albuquerque, New Mexico, USA; PMO, Paleontological Museum Oslo, the University of Oslo, Oslo, Norway; TF, Department of Mineral Resources of Bangkok, Thailand; UHR, Hokkaido University Museum, Sapporo, Japan.

### Geological setting

**Figure 2:**
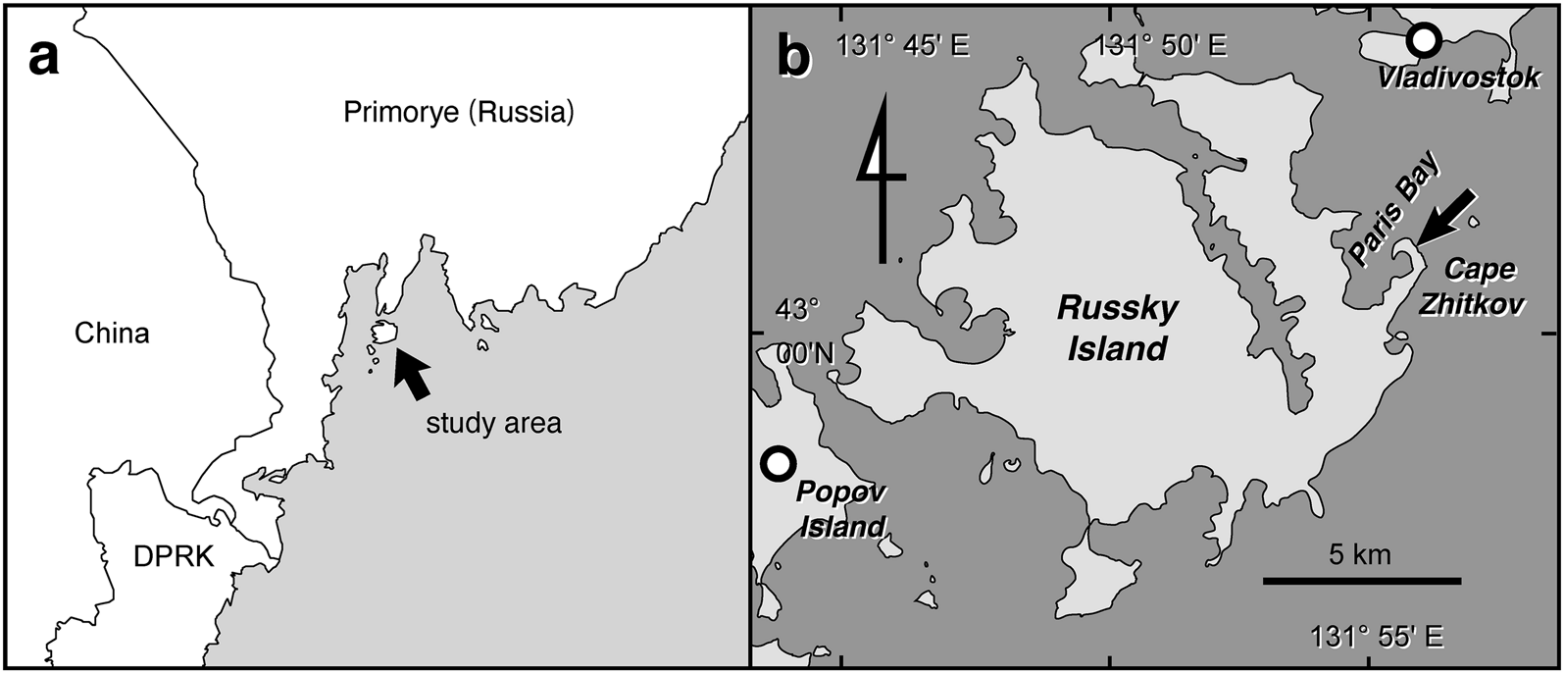
Locality map. **(a)** Location of the study area on Russky Island in the Russian Far East. **(b)** Locality of Cape Zhitkov. The exact site of NSM PV 23854 and 24995 is marked by an arrow. This map is created using Adobe Illustrator ver. 26.1 (https://www.adobe.com/).

**Figure 3:**
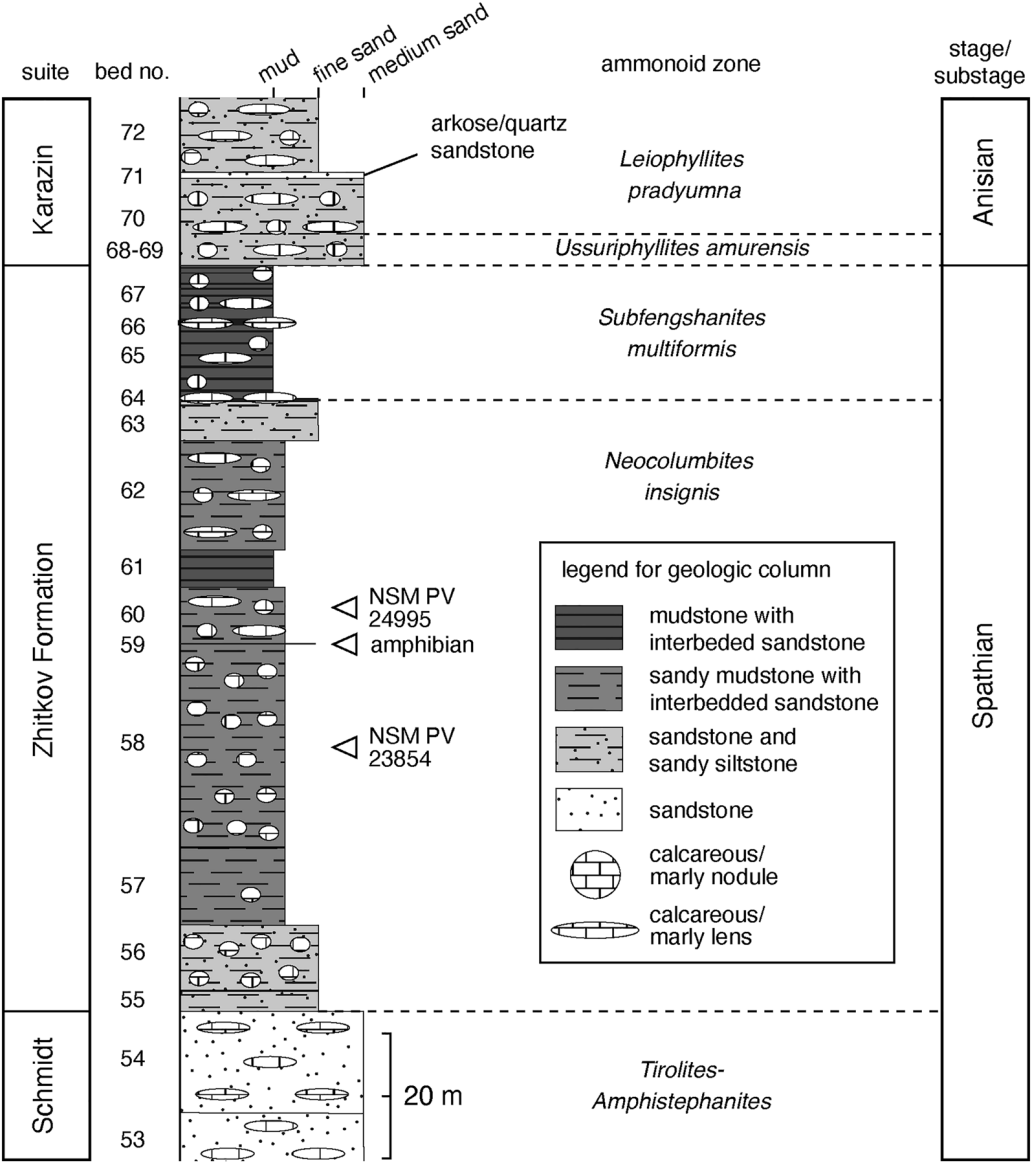
Geological column and ammonoid stratigraphy of the Cape Zhitkov section. Tetrapod horizons are also indicated. Lithology and ammonoid zones were compiled after Zakharov et al.^20,22^ and the personal observations of Y. Shigeta.

The materials were found in calcareous concretions collected in situ from the lower part of the Spathian Zhitkov Formation of Cape Zhitkov, Russky Island, South Primorye (43° 1′ 21.8′′ N, 131° 56′ 3.8″ E; Fig. 2). The Zhitkov Formation conformably overlies the lower Spathian Schmidt Formation (*Tirolites*-*Amphistephanites* ammonoid zone) and shows a fining-upward sedimentary sequence (Fig. 3). The beds of the Zhitkov Formation are numbered from 55 to 68, and the sequence is divided into the *Neocolumbites insignis* (beds 55–63) and *Subfengashites* (formerly *Subcolumbites*) *multiformis* (beds 64–67) ammonite zones, which are overlain by the Anisian Karazin Formation^20^. According to new data obtained from Tchernyschev and Paris bays^21,22^, the uppermost Olenekian *Prohungarites* sp.-*Paranorellina parisi* beds have been proposed for the uppermost part of the Zhitkov Formation. Therefore, the *Prohungarites* sp.-*Paranorellina parisi* beds are not discovered in the Cape Zhitkov section.

The sedimentary facies of the lower Zhitkov Formation represent sandy mudstone with interbedded sandstone, indicating a continental slope depositional environment^23^. A long-snouted temnospondyl amphibian (*Aphaneramma* sp.) has been reported in bed 59^20,24^. There are also occurrences of other Triassic ichthyopterygian fossils in the Russian Far East, including Lower Triassic specimens briefly noted in the literature; however, many of these materials were likely lost without being described^25–27^. In a nearby locality, a durophagous ichthyopterygian, *Tholodus* sp. has been reported in the *Acrochordiceras kiparisovae* zone (Middle Anisian) of the overlying Karazin Formation^22,27^.

Specimens NSM PV 23854 and 24995 are from beds 58 and 60, respectively, both of which belong to the *N. insignis* zone of the lower Zhitkov Formation. This zone best correlates with the *Procolumbites* beds of southern China (Fig. 1), which yielded an early ichthyopterygian, *Chaohusaurus*^7^. Ovtcharova et al.^28^ and Shigeta and Nakajima^29^ considered the *Procolumbites* zone in South China to be “lower” Spathian, whereas Ji et al.^7^ regarded the *Procolumbites* beds as a part of the “middle” Spathian. As there is no widely accepted rule for defining the lower and middle Spathian, this study follows the terminology used in earlier studies^28^; thus, we also regard the *N. insignis* zone in South Primorye as a part of the lower Spathian.

### Systematic paleontology

- **Reptilia Laurenti, 1768**
- **Diapsida Osborn, 1903**
- **Ichthyosauromorpha Motani et al., 2015**
- **Ichthyosauriformes Motani et al., 2015**
- **Ichthyopterygia Owen, 1840**
- **Ichthyopterygia gen. et sp. indet.**

**Figure 4:**
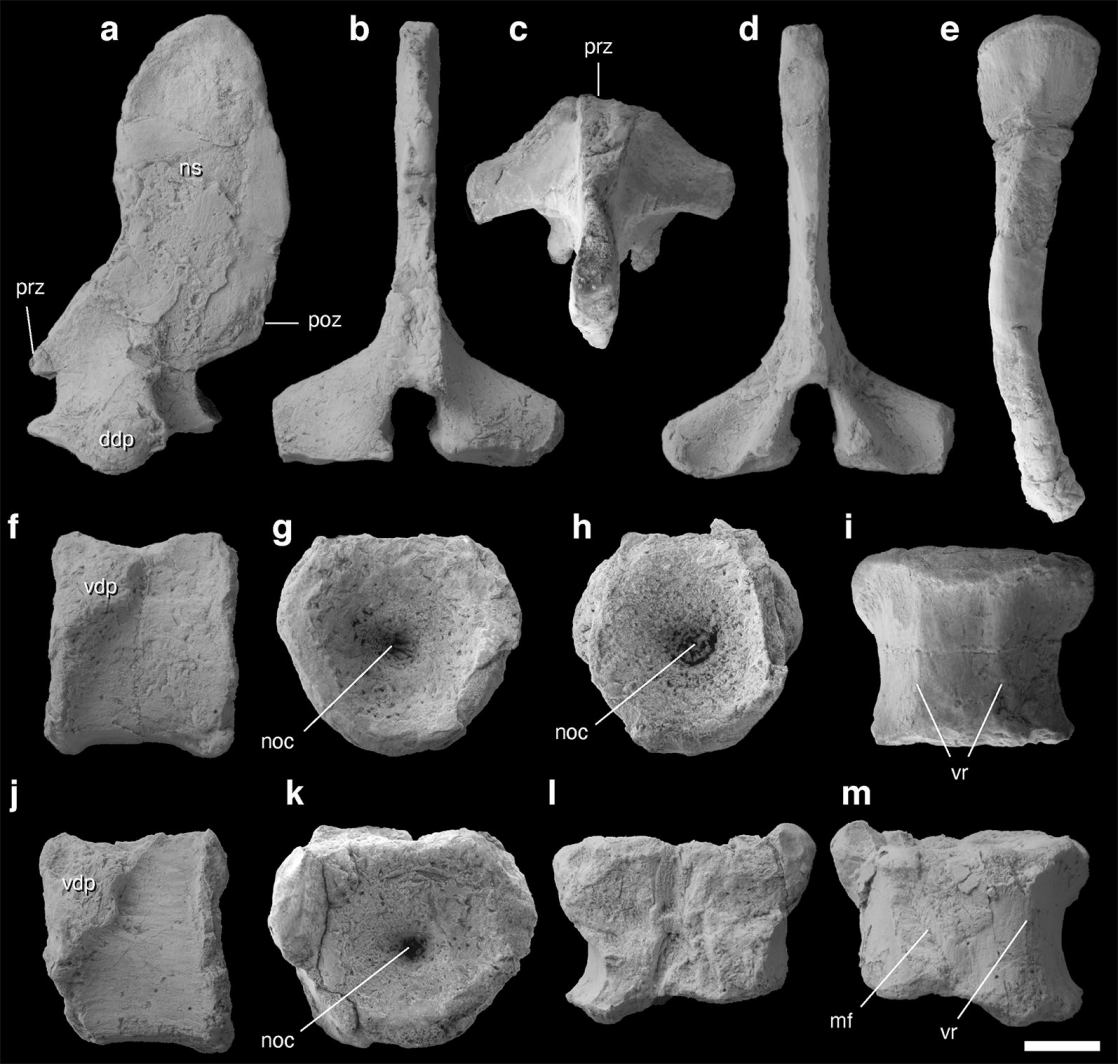
Axial elements of Ichthyopterygia gen. et sp. indet. NSM PV 23854 identified from a single concretion. **(a–d)** Neural arch in (**a**) left lateral, (**b**) anterior, (**c**) dorsal, and (**d**) posterior views. **(e)** Proximal portion of a rib in lateral view. (**f**–**i**) Centrum ‘A’ in (**f**) left lateral, (**g**) anterior, (**h**) posterior, and (**i**) ventral views. (**j**–**m**) Centrum ‘B’ in (**j**) left lateral, (**k**) anterior, (**l**) dorsal, and (**m**) ventral views. *ddp* dorsal half of diapophysis, *mf* microfault, *noc* notochordal canal, *poz* postzygapophysis, *prz* prezygapophysis, *vdp* ventral half of diapophysis, *vr* ventral ridge. Scale bar equals 1 cm.

#### Referred specimen

NSM PV 23854 comprises two nearly complete vertebral centra (‘A’ and ‘B’), which are both likely from the dorsal position of the vertebral column (see Remarks for anatomical identification), neural arch (we include the neural spine while using this term), and rib head. These elements were extracted from a single concretion, and their three-dimensional structure is well preserved, although some parts are missing due to weathering and other post-burial processes. Therefore, the rib shaft and the neural spine rim were reconstructed using glue to merge the fragments in position.

#### Locality and horizon

Bed 58 of the Zhitkov Formation (*Neocolumbites insignis* zone, lower Spathian, Lower Triassic)^20^, exposed along the northern shoreline of Cape Zhitkov, Russky Island, South Primorye, Russia.

#### Description

Viewed laterally, the centra of NSM PV 23854 are approximately square in shape; the height/length is 30.8/25.5 mm in centrum ‘A’ (Fig. 4f) and 32.0/29.5 mm in centrum ‘B’ (Fig. 4j). The anterior and posterior intervertebral joint surfaces of each centrum are almost evenly hexagonal, moderately concave near the anterior and posterior margins, and strongly concave near the center, with the middle portions perforated by a small notochordal canal (Fig. 4g,h,k). In both specimens, the centrum contributes to the ventral half of the diapophysis, and the parapophysis is lost or confluent with the diapophysis. These characteristics suggest that the single-headed rib is attached across the neurocentral suture line and near the intervertebral disk. The anterior margin of the diapophysis is truncated, and the transverse processes laterally project from the anterodorsal corners (Fig. 4i). The precise outline of centrum ‘B’ cannot be observed in the ventral view because it is deformed by a microfault; however, it is approximately square and somewhat constricted at the center owing to anterolateral and posterolateral projections (Fig. 4m). Two longitudinal subparallel ridges are present on the ventral surface of the centra (Fig. 4i,m). The floor of the neural canal is an hourglass-shaped depression (Fig. 4l). The sutural facets of the neural arch are smooth, showing no signs of neurocentral suture fusion (Fig. 4l).

The neural arch possesses posterolaterally-projecting transverse processes (Fig. 4c). The ventral border of the transverse process reaches the neurocentral suture (Fig. 4a). Viewed laterally, the base of the neural arch is convex (Fig. 4a) and matches the neurapophysis of centra ‘A’ and ‘B’; therefore, the neural arch may have belonged to either of the vertebrae. The neural arch narrows at the level of the spinal cord in the lateral view and is antero-posteriorly broad in the neural spine (Fig. 4a). Pre- and post-zygapophyses are poorly preserved because of weathering, but it is likely that the original left and right zygapophyses were not distinct, as in the derived Triassic ichthyosaur *Shastasaurus pacificus* (originally *S. altispinus*)^30^ (Fig. 4b,d). The marginal region of the neural spine also appears to be missing and reconstructed, making it difficult to determine its precise outline. However, it is certain that the neural spine is laterally thin, slightly curved anteriorly, and at least 56.5 mm high when measured vertically from the level of the neurocentral suture line (Fig. 4a–d). According to the reconstructed outline of the neural spine, it was likely not much higher than the above measurement, and thus no more than twice as high as the centrum. The size and shape of the dorsal vertebra is close to that of the dorsal region of *U. hataii *^31^.

The rib fragment expands proximally, with a single-headed proximal end and a longitudinally grooved shaft, similar to the dorsal ribs of *Utatsusaurus*^31^ (Fig. 4e). Although the middle portion of the rib fragment is missing because of weathering, we confirm that the rib shaft is slender and curved (Fig. 4e).

#### Remarks

Deeply biconcave centra are commonly observed in Triassic and Jurassic ichthyopterygians, occasionally with a tiny central perforation^30,31^. Previous research has suggested that the depth of the centrum concavity is shallow in *U. hataii* from the late Spathian^32^; however, further preparation and observation of the holotype (IGPS 95941a) and more recent specimens (NSM PV 20028, 21865, UHR 30691) revealed that the vertebrae are deeply amphicoelous in this species^29^ (also confirmed by personal observation by Y.N.). The neural arch and centrum are rarely fused in ichthyopterygians^33^ and separate in the current material also. In fact, none of these characteristics have been solely defined as a synapomorphy of Ichthyopterygia (or Ichthyosauria). For example, a notochordal canal is a plesiomorphy commonly found in basal tetrapods^34^ and amniotes^35,36^. However, the combination of the above-cited characteristics (lack of neurocentral suture fusion, deep amphicoelous centrum with notochordal canal, indistinct zygapophyses, and extremely cancellous bone) has not been reported for any tetrapods other than Ichthyopterygia. In non-ichthyopterygian ichthyosauriforms (i.e., Nasorostra), the centrum anatomy has not been reported in detail. As the vertebral centrum is not exposed or well preserved in published specimens of *Cartorhynchus lenticarpus* and *Sclerocormus parviceps* (personal observation by Y.N.)*,* Nasorostra is postulated as a candidate; however, the associated rib is gracile as in general ichthyopterygian ribs, and not robust or pachyostotic as in nasorostrans^1,13^. Therefore, we identified NSM PV 23854 as Ichthyopterygia gen. et sp. indet.

Cylindrical (anteroposteriorly long) dorsal centra are found in *Californosaurus*, mixosaurids, and basal ichthyopterygians such as *Chaohusaurus*, *Grippia*, and *Utatsusaurus*, which are all from the Triassic^37^. In contrast, post-Triassic derived lineages and a few large Triassic groups, including *Cymbospondylus* and *Shonisaurus*, generally possess shortened dorsal centra. The ratio of the height of the anterior dorsal neural arch relative to the centrum height is less than 2.0 in NSM PV 23854, and less than that of *Gulosaurus* specimens found in British Columbia (between 2.2 and 2.9)^38,39^ and mixosaurids (between 2.0 and 3.0 or higher)^40^. In addition, the microanatomical characteristics (see “Bone microanatomy and histology” section) of the current specimen are typical of those observed in basal, long-tailed ichthyopterygians^41^. Therefore, NSM PV 23854 likely belongs to the basal lineage, at least according to the above morphology. However, this conclusion is not absolute because simplified zygapophyses, which are observed in the derived lineages of Triassic ichthyopterygians^42^, might be present in NSM PV 23854.

A single articular facet crossing the neurocentral suture is common among the posterior cervical and dorsal vertebrae of Triassic ichthyopterygians. This condition is present in the dorsal vertebra of Triassic forms such as *Utatsusaurus* (personal observation by Y.N.), *Cymbospondylus*, *Shastasaurus, Mixosaurus*, *Besanosaurus*, and *Californosaurus*^30,32,43^. Furthermore, the dorsal vertebral centra of *Cymbospondylus* sp. from the Spathian of Spitzbergen^18^ (originally identified as an ichthyopterygian taxon *Merriamosaurus hulkei*^44,45^*,* which is currently disputed) shows anteriorly truncated diapophysis^18^ as in NSM PV 23854, although the height/length ratio of Spitzbergen *Cymbospondylus* centra is almost 2.0, unlike in NSM PV 23854, which has a height/length ratio of almost 1.0. In addition, the associated rib can be identified as a dorsal rib based on its curved shape^46,47^. Therefore, it is likely that the centra and neural arch of NSM PV 23854 are from the dorsal region.

- **Ichthyopterygia gen. et sp. indet.**

**Figure 5:**
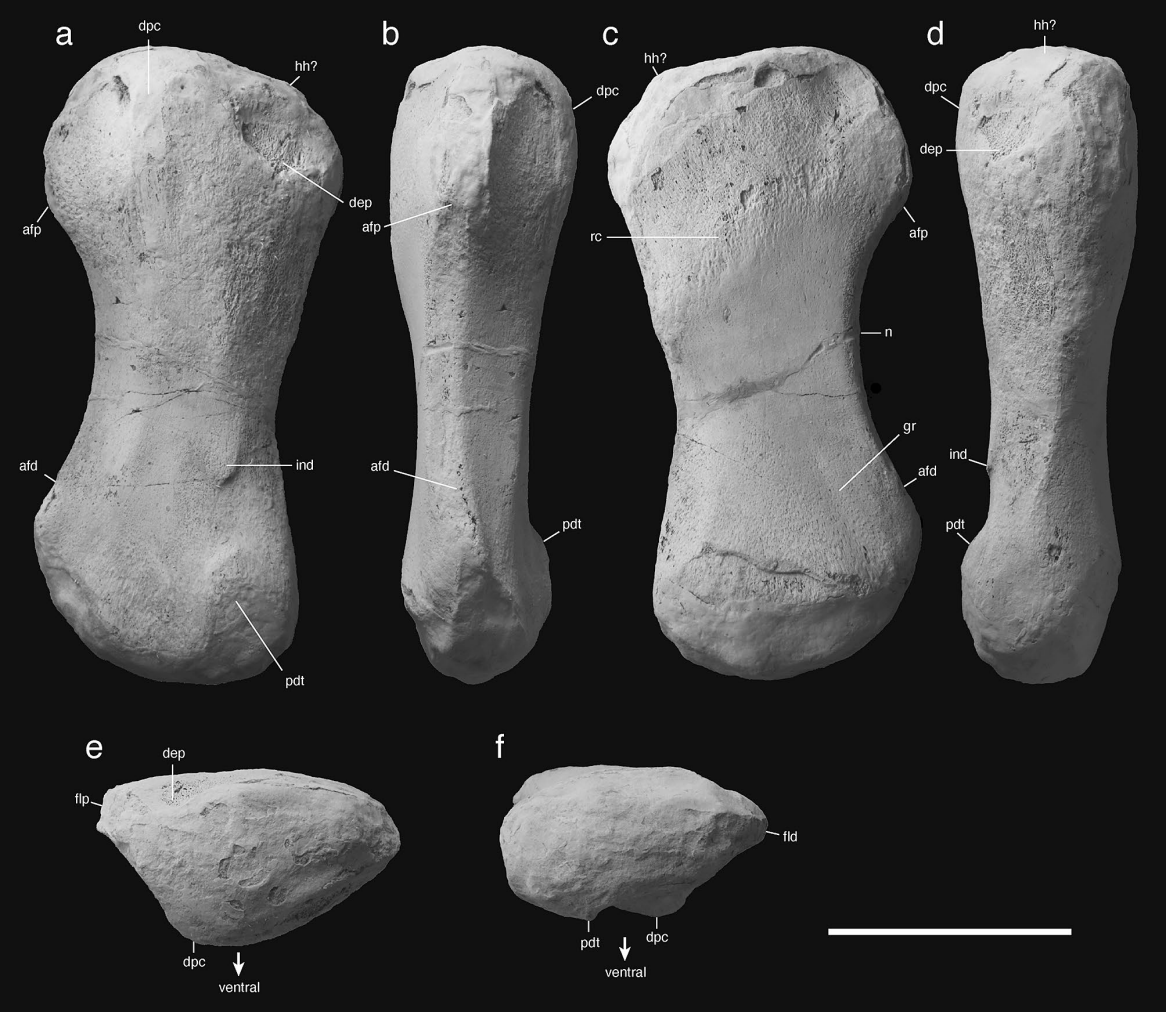
Ichthyopterygia indet. **(a**–**f)** NSM PV 24995, right humerus in (**a**) dorsal, (**b**) anterior, (**c**) ventral, (**d**) posterior, (**e**) proximal, and (**f**) distal views. Scale bar equals 5 cm. *afp* proximal part of anterior flange, *afd* distal part of anterior flange, *dpc* deltopectoral crest, *gr* groove, *hh?* possible humeral head, *ind* indentation, *pdt* postero-distal tuberosity, *rc* rugose crest.

#### Referred specimen

The material NSM PV 24995 is a single limb bone that is morphologically similar to the humerus of the Middle Triassic ichthyosaurian *Cymbospondylus youngorum* LACM DI 157871^48^. The specimen was originally broken into two pieces in the shaft; however, the broken surfaces were fit together to reconstruct the precise total length.

#### Locality and horizon

Bed 60 of the Zhitkov Formation (*Neocolumbites insignis* zone, lower Spathian, Lower Triassic)^20^, exposed along the northern shoreline of Cape Zhitkov, Russky Island, South Primorye, Russia.

#### Description

**Figure 6:**
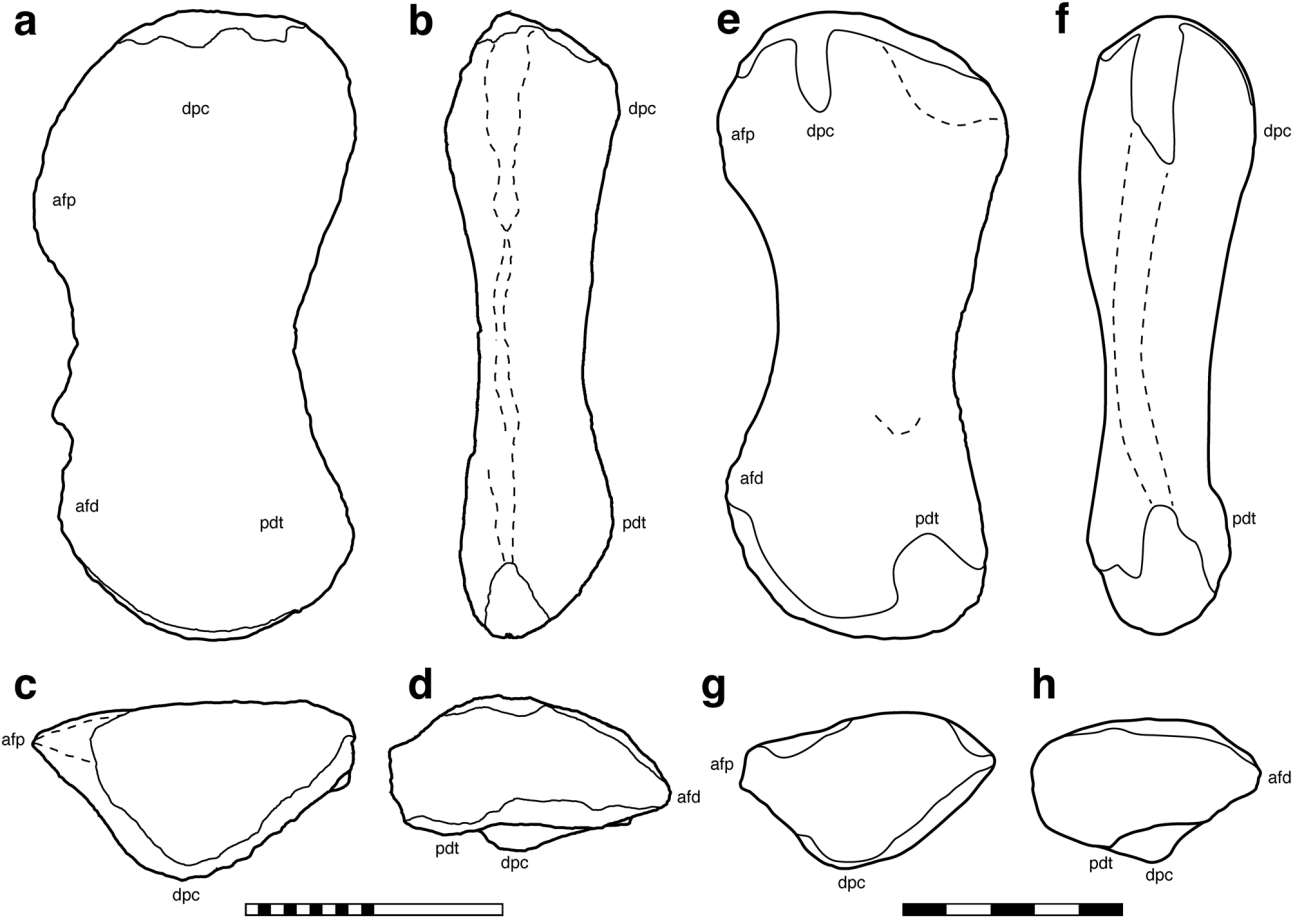
Comparison of humeri in two Triassic ichthyopterygians. (**a–d**) Right humerus of *Cymbospondylus youngorum* LACM DI 157871 in (**a**) ventral, (**b**) anterior, (**c**) proximal, and (**d**) distal views. (**e–h**) Ichthyopterygia gen. et sp. indet. NSM PV 24995 in (**e**) ventral, (**b**) anterior, (**c**) proximal, and (**d**) distal views. Bone contour (thick solid lines), boundary between periosteal and endosteal bones (thin solid lines) and distinct ridges (broken lines) are indicated. Scale bars equal 20 cm in (**a–d**) and 50 mm in (**e–h**). *afd* distal part of anterior flange, *afp* proximal part of anterior flange, *dpc* deltopectoral crest, *pdt* postero-distal tuberosity.

NSM PV 24995 is a limb bone that is approximately dumbbell-shaped and slightly curved (Fig. 5a–d). The specimen is 131 mm long proximodistally; the anteroposterior widths of the smaller and larger ends are 56 and 62 mm, respectively, and the anteroposterior width of the diaphyseal shaft is 39 mm. Both epiphyses exhibit boundaries between the endochondral and periosteal regions, but not many pronounced trochanters, tuberosities, or articular facets. The smaller epiphysis forms a wedge-shape with a projection at a side (Fig. 5f), whereas the larger epiphysis appears uneven triangle (Fig. 5e), both in the end views. A triangular epiphysis has been identified in the humeral proximal end of a basal ichthyosaurian *Cymbospondylus youngorum* from the Anisian, in Nevada, and each of its triangle corners corresponds to anterior margin, posterior margin, and deltopectoral crest, respectively^49^ (Fig. 6). Therefore, this specimen was tentatively identified as a humerus with a triangular proximal end with a shallow deltopectoral process on the ventral side. A small projection in the ventral side of distal metaphysis likely corresponds to the “postero-distal tuberosity” in Motani^50^, which determines the posterior (ulnar) side of the humerus. Thus, we consider NSM PV 24995 as a right humerus. Both anterior and posterior margins of the diaphysis are concave as in humerus of *Cymbospondylus petrinus* and *C. youngorum*^48,49^. The humerus of *Cymbospondylus duelferi* also shows constricted diaphysis; however, it has a much broader distal end than proximal end^49^. The anterior margin of NSM PV 24995 is smoothly curved, forming a very shallow and thick anterior flange (Fig. 5a–c). The anterior flange appears to direct anterodorsally in the proximal view, anteriorly in the midshaft in the anterior view, and somewhat dorsally in the distal view (Fig. 5b,e,f). Such directional change in the flange results in a dome-shaped curvature of the anterior part of the humerus. Similar curvature is known from the convex humeral margin of *Pessopteryx nisseri* from the Spathian (e.g., PMO 229.780, Engelschiøn et al*.*^18^, Fig. 4C3_3_), although Engelschiøn et al*.*^18^ determined this convex margin of the humerus as posterior and the concave as anterior, and one of the proximal projections as “dorsal process”. However, the anatomical orientation of *P. nisseri* humerus lacks consensus partly because this genus is known only from isolated skeletal elements; in fact, Motani^50^ comprehensively compared ichthyopterygian humeri and determined the convex margin of *Pessopteryx* humerus as anterior, and a proximal projection as a deltopectoral crest. Here we follow Motani^50^ and point similarity in the curvature of the anterior flange between NSM PV 24995 and *P. nisseri*, despite the shortened and rounded shape of the latter. The anterior margin of NSM PV 24995 is thickened so that the flange possesses two distinct ridges, while the posterior margin is a sharp, single ridge. The humeral head is indistinct, but a shallow postero-proximal corner might correspond to it (Fig. 5a,c,d). The deltopectoral crest is close to the shaft axis as in *C. petrinus* and *C. youngorum*, but not anteriorly displaced as in *Grippia longirostris* and *Mixosaurus natans*^50^ (Fig. 5a,b,d,e). The ventral aspect of NSM PV 24995 shows a shallow depression near the postero-proximal corner and a small and sharp indentation slightly proximal to the postero-distal tuberosity, but their function is unknown (Fig. 5a,d). In the dorsal surface, a weakly developed, rugose crest distributes in the proximal metaphysis, nearly parallel to the proximal diaphyseal margin, and continues to a shallow tubercle in the posterior margin (Fig. 5c). Also, a very shallow groove develops near the parallel to the antero-posterior region in the dorsal aspect (Fig. 5c).

#### Remarks

Motani et al*.*^50^ categorized ichthyopterygian humeri into morphotypes 1–3, with the exceptional *Cymbospondylus buchseri* (very short humerus with smoothly concave and anteriorly directed anterior flange and complete anterior and posterior shafts), *C. petrinus* (the elongate version of *C. buchseri*), and *Merriamia zitteri* types. Among these types, NSM. PV 24995 shares some characters with type 1 (complete posterior shaft as a basal condition) and 2 (pronounced postero-distal tuberosity as one of the derived conditions). The anterior flange is fundamentally short, but it is present both proximally and distally, not only distally as in Type 3 and *M. zitteri*. Therefore, NSM PV 24995 is most comparable to *C. petrinus* type, although the postero-distal tuberosity is more pronounced than in *C. petrinus*. The function of the rugose crest is unknown, but it could be interpreted as the insertion for dorsal muscles. It is also unknown what the postero-distal shallow groove is for, but it would be a rudimentary entepicondylar groove, which is not common among ichchyopterygians^35^. The concave anterior flange is a symplesiomorphic character in Ichthyosauromorpha found in basal members such as hupehsuchians, *Cartorhynchus* and *Chaohusaurus*^1,50–52^; however, similar condition is also found in more derived taxa including *Cymbospondylus*, *Pessopteryx,* and *Shonisaurus*^1,48,49^. In considering that NSM PV 24995 shares other characters with *Cymbospondylus* and shastasaurids, it is most reasonable when we assume that the current specimen belongs to Ichthyopterygia.

### Bone microanatomy and histology

**Figure 7:**
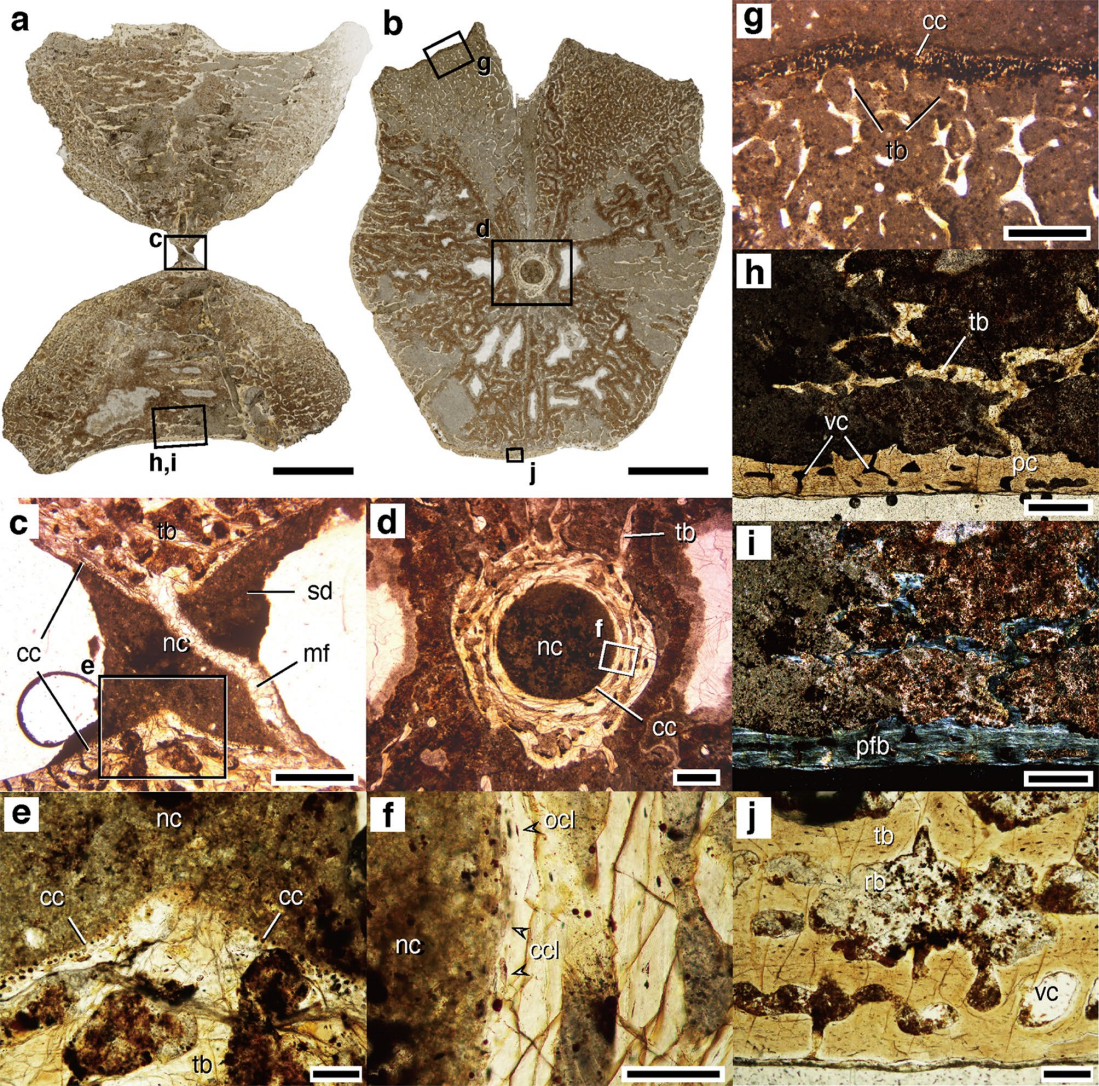
Microanatomy and histology of NSM PV 23854 centra. **(a**,**c**,**e**,**h,i)** Mid-sagittal section of centrum ‘A’: **(a)** entire section, (**c)** close-up photo of growth center, **(e)** calcified cartilage around nutrient canal, and **(h,i)** ventral wall of periosteal cortex, under **(a)** natural, **(h)** plane-polarized, and **(i)** cross-polarized lights. **(b**,**d**,**f**,**g,j)** Mid-transverse section of centrum ‘B’: **(b)** whole section, **(d)** growth center, **(f)** close-up view of bony wall surrounding nutrient canal, **(g)** calcified cartilage on the neurocentral contact surface, **(j)** ventral wall of periosteal cortex under **(b)** natural and **(d,f,g,h)** lights. Enlarged areas **(c**–**j)** are indicated by rectangles in **(a–d)**. *cc* calcified cartilage, *ccl* cartilage cell lacuna, *mf* microfault, *nc* notochordal canal, *ocl* osteocyte lacuna, *pc* periosteal cortex, *pfb* parallel-fibered bone, *rb* resorption bay, *sd* sediment, *tb* trabecular bone, *vc* vascular canal. Scale bars equal 5 mm for (**a,b**), 0.5 mm for (**c,d,g,h,i,j**); 0.1 mm for (**e,f**).

The centra exhibit internal cancellous bone in the endochondral and periosteal domains, the trabeculae of which are widely spaced in the latter. In the mid-sagittal section, the endochondral domain forms an X-shape that expands from the growth center toward the edges of the anterior and posterior intervertebral surfaces (Fig. 7a). The endochondral domain also expands from the growth center toward the right and left neurocentral contact surfaces, as observed in the mid-transverse section (Fig. 7b). The intervertebral surfaces and the neurocentral contact surfaces are covered with a very thin layer of calcified cartilage, which represents the growth front of the endochondral domain, comprised secondary trabecular spongy bone (Fig. 7c,e,g). The periosteal domain comprises a loose network of trabecular bone surrounded by thin (0.2–0.5 mm) periosteal bone (Fig. 7h,i). The trabeculae in the periosteal domain have longitudinal and radial orientations in the sagittal and coronal sections, respectively, evoking a three-dimensional reticular network. The thin periosteal bone comprises longitudinally oriented parallel-fibered bone tissue and is associated with longitudinally oriented and concentrically aligned vascular canals (Fig. 7h–j). The centra are anteroposteriorly perforated in the middle, forming notochordal canals (Fig. 7c,d). Notochordal canals are surrounded by a bony tube lined by a thin layer of calcified cartilage (Fig. 7c–f).

**Figure 8:**
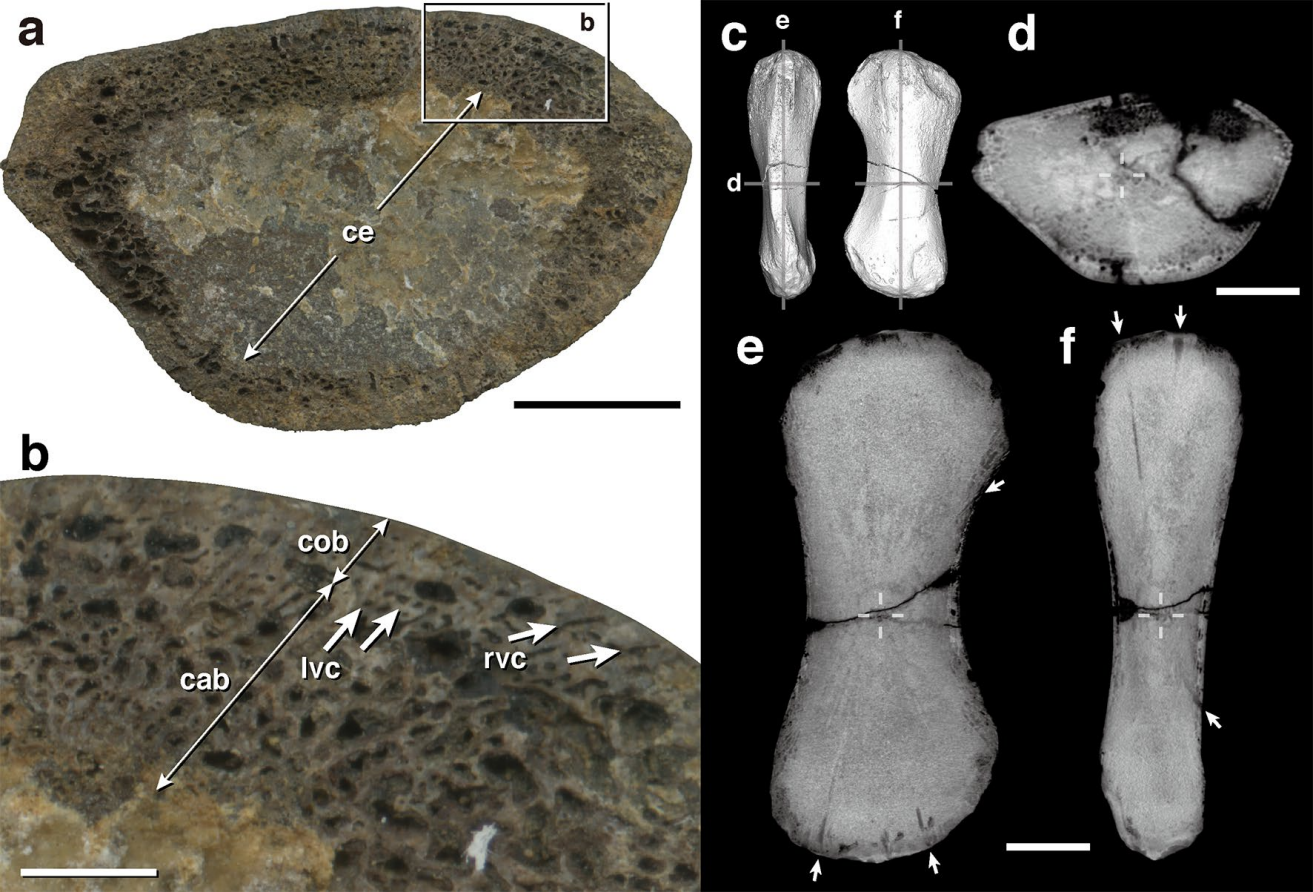
Internal microanatomy of humeral specimen NSM PV 24995. (**a**) Cross-sectional view showing cancellous inner trabecular organization. Intertrabecular spaces around the center are occupied by sedimentary carbonate mineral cementation. **(b)** Close-up view of a superficial area. Enlarged area is indicated by a rectangle in (**a**). **(c–f)** CT scan images. **(c)** Diagram showing sectional positions in (**d**–**f**). **(d)** transverse, **(e)** sagittal, **(f)** sagittal plane. Arrows indicate nutrient canals; broken crosses indicate the position of growth center. *cab* cancellous bone, *ce* cementation, *cob* compact bone, *lvc* longitudinal vascular canals, *rvc* radial vascular canals. Scale bars equal (**a,d**) 1 cm, (**b**) 2 mm, and (**e,f**) 2 cm.

The fracture surface of the mid-diaphysis of the NSM PV 24995 humerus shows an extremely cancellous inner organization with thin trabeculae and a small proportion of peripheral compact cortex (Fig. 8). The inner two-thirds of the cross-section are cemented with carbonate minerals. The outer zone consists of cancellous bone with thin trabeculae and represents the only peripheral 1–2-mm-thick layer of compact bone, but the bone compactness in the internal zone is hardly recognizable from the fracture surface due to the cementation (Fig. 8b). The compact bone is highly vascularized with longitudinal and radial canals, where the former exhibit a circumferential arrangement. The vascular canals are transformed into larger intertrabecular spaces in the internal region. In the computed tomography (CT) scan images, we confirm that the whole internal region is filled with cancellous bone (Fig. 8c–f), and the endochondral domain appears to expand from the growth center toward both ends, making an hourglass shape; the surrounding rest corresponds to the periosteal region (Fig. 8e,f). Nutrient canals run from the growth center to the bone surface, both in periosteal and endochondral domains (Fig. 8e,f).

## Discussion

### Stratigraphic range of early ichthyopterygians

In each of the seven regions with Early Triassic marine reptile fossils, the lowermost occurrence of ichthyopterygian is no older than the early Spathian (*Tirolites*, *Procolumbite*s, *Neocolumbites*, *Columbites*, and the upper part of the *Bajarunia euomphala* beds or (sub) zones), when this unit is present (Fig. 1). In the case of Western USA, the oldest record of Ichthyopterygia is approximately 249 Ma, in the early early Spathian^28,53^, approximately 3 million years after the P–T boundary. Therefore, the diversification and geological radiation of ichthyopterygians occurred quite rapidly because it was not until the P–T boundary that they appeared in a marine environment^54^. Hitherto, large ichthyopterygians that appear to be longer than 5 m have been reported in and above the middle Spathian or are poorly dated^2,16^.

Outside USA, records of middle-sized marine reptiles are possibly from the lower Spathian in Japan (see below). The ammonoid *Columbites* and the biostratigraphically younger *Subcolumbites* are mutually exclusive in the western United States and southern China^55,56^, although these zones are not found in British Columbia^57^ (Fig. 1). Some previous studies have noted that *Utatsusaurus* ranges only within the *Subcolumbites* zone and not in the *Columbites* zone^2,5^, partly because of the misleading definition of *Columbites* and *Subcolumbites* subzones within the *Subcolumbites* zone sensu Bando and Shimoyama^58^, which is clearly not equivalent to the currently accepted middle Spathian *Subcolumbites* zone. To avoid confusion, we refer to the *Subcolumbites* zone sensu Bando and Shimoyama (1974) as the “*Columbites*–*Subcolumbites* zone” in this study.

Several ichthyopterygian materials, including the *U. hataii* holotype (IGPS 95941) and paratype (IGPS 95942), come from the upper part of the “*Columbites–Subcolumbites* zone” (lower–middle Spathian) and “*Arnautoceltites* zone” (upper Spathian) of the Osawa Formation^32,59^. However, some “*Utatsusaurus*” materials have been found in the lower part of the “*Columbites*–*Subcolumbites* zone” in the Osawa Formation of Utatsu-Tatezaki (specimen “nos. A–C”)^32^. Because these fossils are not described or illustrated, their actual affinity is unknown, although they might be comparable to *Utatsusaurus* materials in terms of their size and shape, given that Shikama et al.^32^ assigned them to this genus.

A recent study re-identified several ammonoid fossils from the type section of the Osawa Formation as *Hellenites tchernyschewiensis*, *H. inopinatus*, *Neocolumbites grammi*, *N. insignis*, *Procolumbites ussuriensis*, and *P. subquadratus* in its lower part; therefore, the lower Osawa Formation can be correlated to the *N. insignis* zone of South Primorye^60^. It is possible that Utatsu-Tatezaki, the other ichthyosauromorph-bearing “*Columbites*–*Subcolumbites* zone” locality, which is the type locality of *Utatsusaurus*, also includes *N. insignis* (upper lower Spathian) and *Subcolumbites* (middle Spathian) subzones because no large missing parts have been recognized between the underlying Hiraiso Formation and Osawa Formation^32,61,62^. As evidenced by Smith et al.^3^ and this study, some medium (~ 2.5 m) to large (~ 5 m) ichthyopterygians were present during the early Spathian, shortly after the P–T boundary (approximately 3 Mys^53^), thus, it is not surprising that lower Spathian marine reptiles exist among Japanese collections.

According to the diversity of Hupehsuchia, Nasorostra, and basal species of Ichthyopterygia in the Spathian of southern China, Motani et al.^1^ suggested that Ichthyosauromorpha originated in this area. The diverse ichthyosauromorphs in the Nanlinghu Formation are assumed to represent one of the oldest records of Ichthyosauromorpha (Fig. 1). To date, hupehsuchians and nasorostrans have been reported exclusively in South China; however, rib specimens reported from the Osawa Formation (UMUT MV 31051) show a thickened shaft and a broad head that closely resembles that of a large nasorostran *S. parviceps*^13,46^. Therefore, further investigation of incomplete materials from the lower Spathian in Japan and the Russian Far East are required to better understand the origin of ichthyosauromorph groups.

### Body size and ecology of Early Triassic marine reptiles

Marine reptile body size increased rapidly during the Early Triassic^2,13^. As noted above, the vertebral size of the oldest known ichthyopterygian from *Tirolites* beds reaches ~ 3.5 cm in diameter^3^, which presumably corresponds to the largest vertebra of a ~ 3-m-long ichthyopterygian. Other lower Spathian ichthyopterygians are generally small; *Chaohusaurus* is approximately 1 m long and smaller than the largest known contemporary predatory fish^2,5^. *Thaisaurus chonglakmanii* is known from a single specimen TF 2454, consisting of an almost complete skull and incomplete postcranial skeleton, and is close to *C. geishanensis* in size and shape^4,37^. The cranial length of *T. chonglakmanii* is ~ 10 cm, and the estimated body length is 53–84 cm based on the skull/body proportion in embryonic and adult *C. geishanensis*^4,5^. Although the growth stage of the *T. chonglakmanii* holotype has not been estimated precisely, the ossification stage of carpal bones in the only specimen is close to that of adult *C. geishanensis *^4,63^. Thus, *T. chonglakmanii* is likely a small ichthyopterygian less than 1 m long.

The new vertebrae from the Russian Far East are similar in size to the mid-dorsal vertebrae of *U. hataii* UHR 30691, which reached a total body length (TL) of 2.6 m^31^, although the precise TL of NSM PV 23854 is hard to predict. The body size of NSM PV 24995 is not known either; however, the humrus length (HL) is approximately twice that of *U. hataii,* whose humeri are 61 mm and 66 mm long based on UHR 30691, neglecting post-burial deformation (see Motani et al.^31^ regarding the necessity of retro-deformation). Furthermore, linear regression analysis on the relationship between HL and TL (both in meter) by Sander et al.^48^ found a relationship of log_10_(TL) = 1.599 (s.e. = 0.038) + 1.023 (s.e. = 0.035) × log_10_(HL), with R^2^ = 0.9801^48^. Accordingly, we speculate that a suitable TL of NSM PV 24995 (HL = 0.131 =  ~ 10^–0.88^) is approximately 5.0 (~ 10^0.7^) m. An upper Spathian ‘ichthyosaur humerus’ NMMNH P-65886 is even approximately twice as long as NSM PV 24995^2^. As noted above, the identity of this specimen is controversial^2,13^, but the original identification is convincing because of a significant dorsoventral thickness, distinct proximal and distal ends, and unambiguous deltopectoral crest and notched anterior flange^2^, as in NSM PV 24995. If we regard NMMNH P-65886 as a humerus, ichthyopterygians likely reached a gigantic body length of ~ 11 m until the middle-late Spathian^2^. The increasing body size trend of Ichthyopterygia is well recognized in the *Grippia* Niveau (lower Spathian) to Lower Saurian Niveau (upper Spathian) of Spitzbergen^16,18^. The size of ichthyopterygians possibly reached ~ 3 m in the age of lower Spathian *Tirolites* zone as evidenced by a vertebral centrum measuring ~ 3.5 cm in diameter^3^, which is slightly larger than the largest *Utatsusaurus* centra (~ 3 cm in NSM PV 20028, TL =  ~ 2.6 m). In Hupehsuchia and Sauropterygia, a few fossils from the Early Triassic exhibit exceptionally large body sizes, although these materials are not well dated^64–66^. Overall, the trend toward gigantism in Ichthyopterygia became obvious before the end of the Spathian around the world, and the discovery of early Spathian large (total body length >  ~ 5 m) marine reptiles from the eastern margin of Panthalassa (this study) suggests that such gigantism started, perhaps globally, before the middle Spathian.

The body sizes of osteichthyan fish, on the other hand, drastically increased across the Middle–Late Permian boundary, but exhibited no significant change across the P–T boundary^67^. In the Smithian, marine predator niches were occupied by chondrichthyans, temnospondyl amphibians, and osteichthyan fishes, including the 1.85-m-long *Birgeria americana* from Nevada^2,68^. Subsequently, the body size of osteichthyan fish decreased across the Early–Middle Triassic boundary, shortly after large marine reptiles appeared in the fossil record^67^. This size reduction in Osteichthyes could be considered as the result of their competition with marine reptiles.

The gigantism of ichthyopterygians during the Spathian might be due to their physiological advances over fish, including rapid growth and a high metabolic rate, as evidenced by high vascularization in parallel-fibered bone tissue or fibrolamellar complex^69,70^. Furthermore, oceanic environments underwent fluctuating anoxic to oxygenated conditions during the Early Triassic, as suggested by many authors^71,72^; however, air-breathing marine reptiles were not likely as severely disturbed by oceanic anoxia-dysoxia as fish, at least in terms of respiration (although it should be noted that some osteichthyans are capable of lung breathing as well as gill breathing). Among reptiles and other amniotes, the loose spongious organization of bones is frequently observed in highly advanced swimmers and divers, such as modern whales. In contrast, increased bone mass is common among semi-aquatic forms like sea otters and poorly active shallow divers like sirenians^73^. The limb bones filled with loose spongious tissue have been reported from the marine turtles, especially the pelagic and deep-diving species *Dermochelys coriacea*^74–76^. Ichthyopterygian bone compactness has a considerable variation^41,70^, and the current specimens are particularly cancellous. In addition, radial nutrient canals in the endochondral domain are also observed in marine turtles *Dermochelys* and *Archelon*^76^, as well as in *Pessopteryx*^77^ and derived plesiosaurs^78^. Therefore, the bone microanatomy in NSM PV 23854 and NSM PV 24995 suggests that these reptiles were already highly adapted to an aquatic lifestyle, supporting the hypothesis that the ichthyosauromorph ancestor had already adapted very rapidly to the oceanic environment by the early Spathian^54^. The sedimentary facies of marine reptile-bearing beds in the Lower Zhitkov Formation (sandy mudstone with intercalated turbidite sandstone) represent a continental slope depositional environment^23^, and the paleogeographic position of South Primorye faced the western Panthalassa ocean in the Early Triassic^55^. These support the observation that the ichthyopterygians reported in this study were well adapted to life in the open ocean; however, it is also possible that the carcasses were transported from shallow marine by turbidity currents.

## Methods

Thin sections were prepared from centrum specimens (NSM PV 23854) using a standard procedure described previously^79^. Transverse and mid-sagittal sections were taken from centra ‘a’ and ‘b’ respectively, so that the sectional planes passed through the growth center. Thin sections were observed under normal and cross-polarized transmitted light using a Leica DMLP polarizing microscope. Thin-section photographs were taken macroscopically using an EPSON GT-X980 image scanner and microscopically using a Leica DFC420 digital camera attached to the microscope. A long bone (NSM PV 24995) was originally broken into two pieces in the shaft. The shaft was reconstructed using epoxy adhesive after the sectional plane was photographed for microanatomical observation using an image scanner (EPSON GT-X980). Reconstructed specimen NSM PV 24995 was CT-scanned using inspeXio SMX-225CT FPDHR micro-focus X-ray system (Shimadzu Co.) in National Museum of Nature and Science (Tsukuba, Japan), 225 kV, 70 μA, 167.45 μm/voxel.

## Data Availability

All data generated or analyzed during this study are included in this published article.
